# Use of Phenol as a Local Anaesthetic for Adult Grommet Insertion in Resource-Limited Settings: A Preliminary Report

**DOI:** 10.1155/2019/2893418

**Published:** 2019-08-01

**Authors:** AbdulAkeem Adebayo Aluko

**Affiliations:** Department of Otorhinolaryngology, Bayero University/Aminu Kano Teaching Hospital, Kano, Nigeria

## Abstract

**Background:**

Grommet insertion is one of the most commonly performed minor surgical procedures in otolaryngological practice. For such minor procedures in the outpatient, local anaesthetics are preferred; this is even more so in adults especially for grommet insertion. This study described our experience with the use of phenol as a local anaesthetic agent for grommet insertion in adults.

**Methods:**

Phenol was used as a local anaesthetic agent that was applied topically for grommet insertion in adult patients as outpatient procedures between January and September 2018 in two tertiary hospitals. Data collected were analyzed using the Statistical Package for Social Sciences (SPSS IBM) version 23.0 computer software.

**Results:**

Nineteen ear drums were operated in patients aged between 20 and 52 years. No pain or discomfort was reported by 89.5% and 94.7% had no bleeding. There was no vertigo in all the cases that completed the procedures.

**Conclusion:**

This preliminary result shows that the use of phenol as a topical local anesthetic is simple, safe, and effective especially in resource-limited environments.

## 1. Introduction

Of the many minor eardrum procedures requiring general or local anaesthesia, myringotomy with or without the insertion of grommet is one of the most common procedures performed by otolaryngologists today. Majority of these grommet insertions are completed using general anaesthesia, especially in the paediatric patients. However, local anaesthesia is preferred in adult cases most especially in the outpatient or office setting [1–3].

The use of general anaesthesia for myringotomy with or without the insertion of grommet is associated with restlessness, disorientation, airway irritation, cardiac depression, and emergence agitation with most patients receiving general anaesthesia also requiring additional analgesia due to postoperative pain [4]. Topical application of a local anesthetic agent can induce adequate insensibility to pain and therefore can avoid the use of general anesthesia or invasive infiltration techniques for myringotomy or the insertion of a tympanostomy tube [5].

The use of local anaesthetics began in 1884 with the use of 10% cocaine in alcohol by Emmanuel Zaufel [6]. Since then, many local anesthetics have been used, such as Lignocaine, cocaine, Eutectic Mixture of Local Anaesthetics (EMLA), tetracaine base (dissolved in isopropyl alcohol or dimethyl sulphoxide), acid carbolic liquefact, grays fluid (cocaine and aniline oil), Blegvad solution (cocaine and salicylic acid), and Bonnisan liquid (phenol, methanol, and cocaine) [6–8]. However, each is not without its advantages and disadvantages [6, 7, 9, 10]. The optimal local anesthetic for myringotomies or the insertion of tympanostomy tubes in adults should be easy and rapid to use, painless during application, reversible, and inexpensive and should provide good anesthesia and not cause any long-term damage to the tympanic membrane (TM) [5–7].

Topical phenol as a local anesthetic is an alternative [3]. Phenol was first used by Lister as an antiseptic in 1867 but its use for eardrum anesthesia was first advocated by Lloyd Storrs in 1956 [6]. Phenol is also known as phenyl alcohol having the chemical formula as C6H5OH and it is called carbolic acid due to its acid properties. Its other properties of interest are bacteriostatic in 0.2%, bactericidal in 1.0%, and fungicidal in 1.3% [2]. It aids faster creation of tympanic membrane incision and decreases postoperative bleeding through its tissue vaporizing chemical cauterization effect with negligible toxicity if given in minute amount [2]. Despite its advantages, it is a caustic agent and could be toxic [8, 11] but it is generally safe if properly used and handled [2, 6, 8].

Our experience using phenol for anaesthetizing the ear drum for the insertion of grommet in adults is described.

## 2. Methods

This was a descriptive cross-sectional study using convenience sampling to select subjects/participants needing grommet insertion for various indications. The study was carried out on adult patients as outpatient procedures between January and September 2018 in two tertiary hospitals (Aminu Kano Teaching Hospital, Kano, Nigeria, and Rasheed Shekoni Federal University Teaching Hospital, Dutse, Nigeria).

The anesthetic agent used was phenol in aqueous form of 25% solution that was topically applied. And the instruments consist of a set of Hartmann's ear speculums, suction machine set at low pressure (Easycare portable suction machine model), micro ear suction nozzles of various sizes (Fisch Suction tube, angular, with grip plate), Jobson Horne probe, myringotomy knife (Medtronic model), micro ear crocodile forceps, grommet inserter (Saharan surgical model), and otomicroscope (Zeiss Opmi Pico) or 0 degree otoendoscope (Vansari optical telescope 2.7 mm). After explaining the procedure clearly to the patient and obtaining a written/informed consent for it, the patient was positioned lying down on the couch with the treated ear up towards the operator and the appropriate size ear speculum was carefully inserted to examine the TM. The ear canal was cleaned of any wax or debris to get the full view of TM. A Jobson Horne's probe was dipped into the phenol solution to pick up phenol solution and then lowered very carefully down the speculum and inner part of external ear canal onto the site for the myringotomy incision at the anterioinferior quadrant without touching the speculum or canal. Blanching was used to confirm that this area of the TM was anaesthetized and ready for surgery. A linear incision was made at the anaesthetized area on the TM, effluent was sucked out if present, and grommet inserter was used to place the grommet on the TM with the aid of a microscope or an endoscope.

The procedure was done as briefly as possible as the effect of the phenol anesthesia lasts about 15-20 minutes [2]. All the procedures were done on an outpatient basis. All patients were allowed to go home or to work immediately after the operation. They were followed-up thereafter.

During the procedure, bleeding and the patient's experience of pain, discomfort, sting sensation, vertigo, and duration of the procedure were verified and documented. The procedure was discontinued if the patient felt severe pain and/or became uncooperative.

International Business Machines [IBM]. Statistical Package for Social Sciences [SPSS] for Windows, Version 23.0 software [Armonk, NY: IBM Corp] was used for statistical analysis and results were summarized using frequencies, percentages, and graphic representations. Ethical clearance was obtained from the Institutional Ethical Review Committee of the Hospital. The study protocol was explained to the participants. Subsequently, informed consent was obtained and respondents appended their signatures/thumbprints on the consent form. Confidentiality of the respondents was strictly ensured. The study was carried out according to the Declaration of Helsinki [12].

## 3. Results

**Figure 1:**
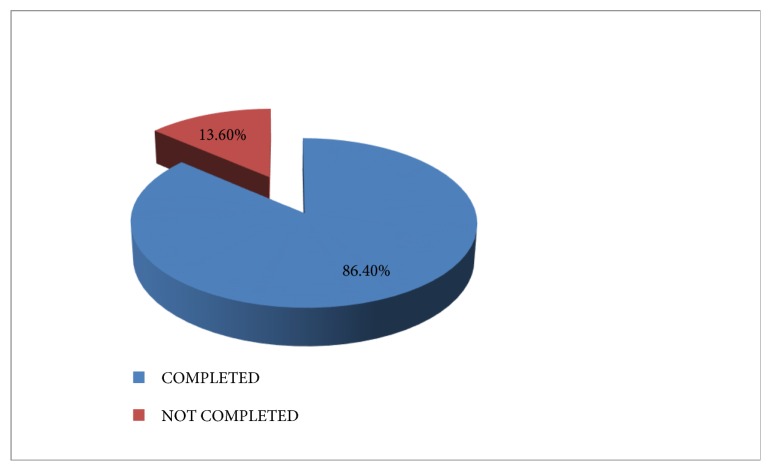
COMPLETION OF PROCEDURE.

The procedure was performed on a total of 22 TMs in 19 patients that were recruited for the study. However, 3 (13.6%) TMs did not complete the procedure as it was discontinued due to apprehension and lack of cooperation while 19 TMs (86.4%), composed of 12 TMs in males and 8 TMs in females, completed the procedure as shown in Figure 1.

The age ranged from 20 to 52 years (median 34.5 years).  Table 1 shows the sociodemographic characteristics of the participants.

The procedure was unilateral in 13 and bilateral in 3 as shown in Table 2.

**Figure 2:**
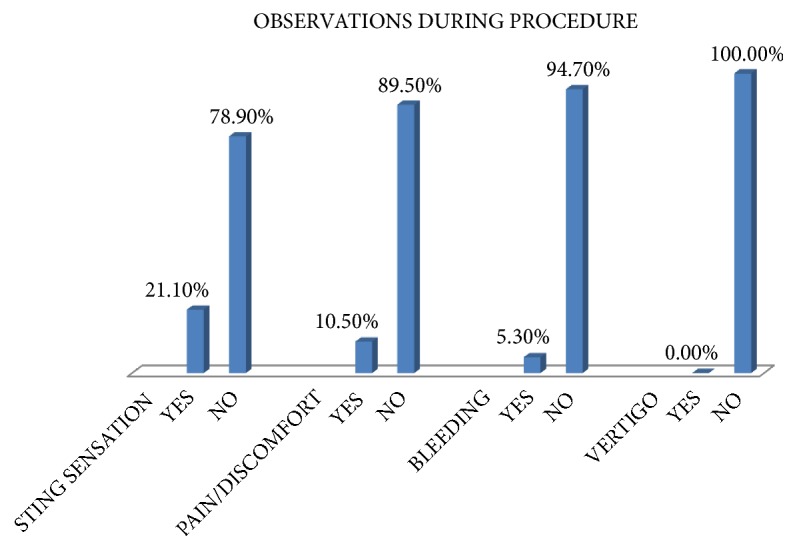


Amongst those who completed the procedure, 4 (21.1%) experienced sting sensation, 1 (5.3%) had minimal bleeding and 2 (10.5%) had discomfort, but none had vertigo as shown in Figure 2.

## 4. Discussions

Since topical application of a local anesthetic agent induces adequate insensibility to pain thereby avoiding the use of general anesthesia for the insertion of a grommet [5], it is often preferred in resource-constrained environments such as developing countries where anesthesiologists may be in short supply. Furthermore, topical phenol was also found to have good use in the relief of postoperative pain when used as an adjuvant anaesthetic in children undergoing tympanostomy tube insertion under inhalational general anaesthesia [13]. Majority (89.5%) of the patients in this study experienced neither pain nor discomfort during the procedure or in the postoperative period, similar to findings by other authors [2, 9, 14–16]. However, it is an essential realization that the anaesthetic effect of phenol lasts for about 15-20 minutes [2]; thus, any procedure lasting more than this period may be associated with pain. The brief duration of the procedure (average of 12 minutes) in this study may have contributed to a lack of pain in the patients. Therefore, it is important to have ready the instruments and prosthesis so as to avoid any delay which may prolong the surgery and wear out the anaesthesia [2]. Also, the pressure of aural speculum and instrument insertion may explain the discomfort/pain experienced by few.

**Figure 3:**
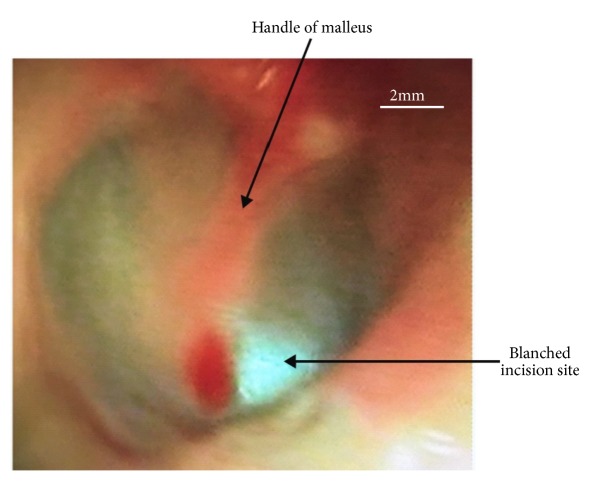
Shows blanching effect of the tympanic membrane by phenol.

**Figure 4:**
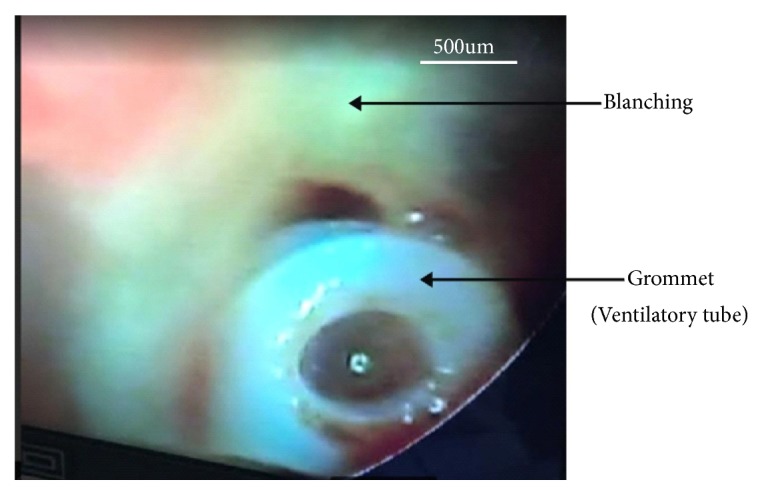
Shows grommet in situ.

Studies by Liston et al. [17] and Sing [2] documented that phenol applied topically to the tympanic membrane is a useful hemostatic agent for myringotomy and insertion of tympanostomy tubes. This supports the finding of no bleeding in 94.7% of the participants who completed the procedure in this study. The reason may be that the blanching effect that occurs (as shown in Figures 3 and 4), due to coagulative necrosis on phenol application, as soon as it touched the TM destroys the blood vessels at that site making the procedure relatively bloodless and therefore less complication of bleeding.

Various local anaesthetics have been used for myringotomy with or without grommet insertion but some were abandoned due to the side effect of severe vertigo that they cause: cocaine, tetracaine, Bonnisan liquid, and lidocaine inclusive [1, 6]. However, this study did not record any patient that experienced dizziness.

Phenol is a highly toxic chemical topically and otologists need to have a healthy respect for the dangers associated with its use [11]. Despite the possible safety issues with phenol as documented in the Globally Harmonised System of Classification and Labelling of Chemicals (H301, H314, H331, H341, and H373), there does not appear to be any evidence of patient injury using phenol topical anaesthesia to the TM [8]; thus, when used in an appropriate manner it can be considered safe [6]. Yet, topical phenol applied to the TM should be used with caution and it remains a potentially dangerous substance if used incorrectly [8]. As in the use of all local anaesthetics, patient selection is very important in the use of topical phenol and some patients will not tolerate the procedure despite adequate local anaesthesia; therefore anxious and uncooperative patients should be avoided. In this study, 13.6% of participants refused to complete the procedure.

## 5. Conclusion

The use of topical phenol gave adequate anaesthesia for grommet insertion in adults with a minimal risk of bleeding and no vertigo in this study. This preliminary result shows that the use of phenol as a topical local anesthetic is simple, safe, and effective especially in resource-constrained/limited environments like developing countries where general anaesthesia may be expensive and/or not readily available. Though phenol is a highly toxic chemical, when used in an appropriate and correct manner with good patient selection, it can be considered a safe and valuable tool. Further studies with larger samples are recommended.

## Figures and Tables

**Table 1:** Sociodemographic profile of participants.

Parameters	Frequency (n = 19)	Percent (%)
*Age (years)*		
** **20-29	10	52.6
** **30-39	6	31.6
** **40–49	2	10.5
** **50–59	1	5.3
*Gender*		
** **Male	12	63.2
** **Female	7	36.8
*Religion*		
** **Islam	17	89.5
** **Christianity	2	10.5
*Ethnicity*		
** **Hausa	16	84.2
** **Igbo	1	5.3
** **Yoruba	2	10.5
*Occupation of caregiver*		
** **Unemployed	1	5.3
** **Housewife	3	15.8
** **Farming	5	26.3
** **Trader	4	21.1
** **Civil servant	4	21.1
** **Others	2	10.5
*Educational status of caregiver*		
None	3	15.8
Primary	9	47.4
Secondary	2	10.5
Arabic/Islamic	5	26.3

**Table 2:** Laterality of procedure on participants.

Parameters	Frequency (n = 19)	Percent (%)
*Unilateral*		
** **Left side	4	21.1
** **Right side	9	47.4
*Bilateral*	6	31.5

## Data Availability

The data used to support the findings of this study are available from the corresponding author upon request.
